# Nanobody engineering for SARS-CoV-2 neutralization and detection

**DOI:** 10.1128/spectrum.04199-22

**Published:** 2024-02-16

**Authors:** Liina Hannula, Suvi Kuivanen, Jonathan Lasham, Ravi Kant, Lauri Kareinen, Mariia Bogacheva, Tomas Strandin, Tarja Sironen, Jussi Hepojoki, Vivek Sharma, Petri Saviranta, Anja Kipar, Olli Vapalahti, Juha T. Huiskonen, Ilona Rissanen

**Affiliations:** 1 Institute of Biotechnology, Helsinki Institute of Life Science (HiLIFE), University of Helsinki, Helsinki, Finland; 2 Department of Virology, Medicum, Faculty of Medicine, University of Helsinki, Helsinki, Finland; 3 Department of Physics, University of Helsinki, Helsinki, Finland; 4 Department of Veterinary Biosciences, University of Helsinki, Helsinki, Finland; 5 Department of Tropical Parasitology, Institute of Maritime and Tropical Medicine, Medical University of Gdansk, Gdynia, Poland; 6 Institute for Molecular Medicine Finland (FIMM), Helsinki Institute of Life Sciences (HiLIFE), University of Helsinki, Helsinki, Finland; 7 VTT Technical Research Centre of Finland Ltd., Espoo, Finland; 8 Laboratory for Animal Model Pathology, Institute of Veterinary Pathology, Vetsuisse Faculty, University of Zurich, Zurich, Switzerland; 9 Department of Infection Biology and Microbiomes, Institute of Infection, Veterinary and Ecological Sciences, University of Liverpool, Liverpool, United Kingdom; 10 HUSLAB, Helsinki University Hospital, Helsinki, Finland; Emory University School of Medicine, Atlanta, Georgia, USA

**Keywords:** SARS-CoV-2, nanobody, virus neutralization, protein engineering, diagnostics

## Abstract

In response to the ongoing COVID-19 pandemic, the quest for coronavirus inhibitors has inspired research on a variety of small proteins beyond conventional antibodies, including robust single-domain antibody fragments, i.e., “nanobodies.” Here, we explore the potential of nanobody engineering in the development of antivirals and diagnostic tools. Through fusion of nanobody domains that target distinct binding sites, we engineered multimodular nanobody constructs that neutralize wild-type SARS-CoV-2 and the Alpha and Delta variants at high potency, with IC_50_ values as low as 50 pM. Despite simultaneous binding to distinct epitopes, Beta and Omicron variants were more resistant to neutralization by the multimodular nanobodies, which highlights the importance of accounting for antigenic drift in the design of biologics. To further explore the applications of nanobody engineering in outbreak management, we present an assay based on fusions of nanobodies with fragments of NanoLuc luciferase that can detect sub-nanomolar quantities of the SARS-CoV-2 spike protein in a single step. Our work showcases the potential of nanobody engineering to combat emerging infectious diseases.

**IMPORTANCE:**

Nanobodies, small protein binders derived from the camelid antibody, are highly potent inhibitors of respiratory viruses that offer several advantages over conventional antibodies as candidates for specific therapies, including high stability and low production costs. In this work, we leverage the unique properties of nanobodies and apply them as building blocks for new therapeutic and diagnostic tools. We report ultra-potent SARS-CoV-2 inhibition by engineered nanobodies comprising multiple modules in structure-guided combinations and develop nanobodies that carry signal molecules, allowing rapid detection of the SARS-CoV-2 spike protein. Our results highlight the potential of engineered nanobodies in the development of effective countermeasures, both therapeutic and diagnostic, to manage outbreaks of emerging viruses.

## INTRODUCTION

Antibody-based products comprise some of the most successful diagnostic and therapeutic tools developed for managing the COVID-19 pandemic, ranging from rapid antigen tests for SARS-CoV-2 infection (1, 2) to neutralizing monoclonal antibodies (mAbs) used to treat individuals at risk of severe COVID-19 symptoms (2, 3). Neutralizing antibodies against SARS-CoV-2 primarily target the Spike (S) protein (4, 5), a glycoprotein mediating host-cell recognition and viral entry (6). SARS-CoV-2 spikes are homotrimers, with each chain consisting of receptor-binding (S1) and fusogenic (S2) subunits (7). S1 contains the receptor-binding domain (RBD) that mediates binding to the primary cellular receptor of SARS-CoV-2, angiotensin-converting enzyme 2 (ACE2) (6, 8, 9). Following receptor binding, S2 subunit, a class I fusion protein, is activated by proteolytic cleavage and mediates fusion of the viral and cell membranes, delivering viral RNA to the cytoplasm (8, 10).

Due to its key role in initiating infection, the SARS-CoV-2 spike is the primary target of vaccines (11) and monoclonal antibody therapy (2). The continued efficacy of these powerful approaches is challenged by the emergence of SARS-CoV-2 variants of concern (VOCs) that display multiple amino acid substitutions in the S-protein (12–14). Following the spread of variants Alpha (B.1.1.7), Beta (B.1.351), and Delta (B.1.617.2) (15–20), Omicron (B.1.1.529) became the dominant circulating variant in 2022, with new Omicron sub-variants still emerging (21). VOC amino acid changes, including a substitution at E484 found in Beta and Omicron, can reduce neutralization by antibodies raised against the “wild-type” SARS-CoV-2 (titled B.1 or Wuhan-Hu-1) (12, 15, 16, 19, 22). Current efforts to mitigate the effects of immune escape on antibody-based COVID-19 countermeasures include the use of antibody cocktails (23, 24) and the development of new antibody-based products, including camelid single-domain antibody fragments (“nanobodies”) (25).

In contrast to traditional mAbs, nanobodies are small (~15 kDa) and offer advantages including nebulized delivery and scalable, cost-effective production in bacterial expression systems (26, 27). During the COVID-19 pandemic, antiviral nanobodies have garnered significant interest, resulting in the discovery and structural characterization of several SARS-CoV-2-neutralizing nanobodies (27–37). Furthermore, as nanobodies comprise self-contained modules, they can be engineered into fusion proteins with enhanced properties. Pioneering studies have started to chart the potential of engineered nanobodies as virus inhibitors (38, 39), but diagnostic applications are largely unexplored. While RT-qPCR remains the gold standard for clinical diagnosis, rapid diagnostic tests designed to detect viral antigens with conventional antibodies (40) are extensively applied in nonhospital settings. We envision that similar antigen tests could be developed using nanobodies.

Here, we apply protein engineering to produce nanobody fusions for enhanced neutralization and as components for a rapid antigen test. Trimodular fusions of selected nanobodies showed up to a 1,000-fold enhancement of the *in vitro* neutralization efficiency against wild-type SARS-CoV-2 as compared to the reported efficiencies of constituent nanobodies (28–30, 41). Nanobody fusions were further engineered to produce proof-of-concept for a novel diagnostic assay, which applies nanobodies fused to fragments of a split signal molecule, NanoLuc luciferase (42–45), and allows the detection of picomolar concentrations of SARS-CoV-2 spike protein in a single step. Overall, our study shows the potential for engineered nanobodies as antiviral and diagnostic agents, which we envision can offer affordable and scalable countermeasures during future outbreaks of emerging viral diseases.

## RESULTS

### Structure-guided design of multimodular nanobodies

**Fig 1:**
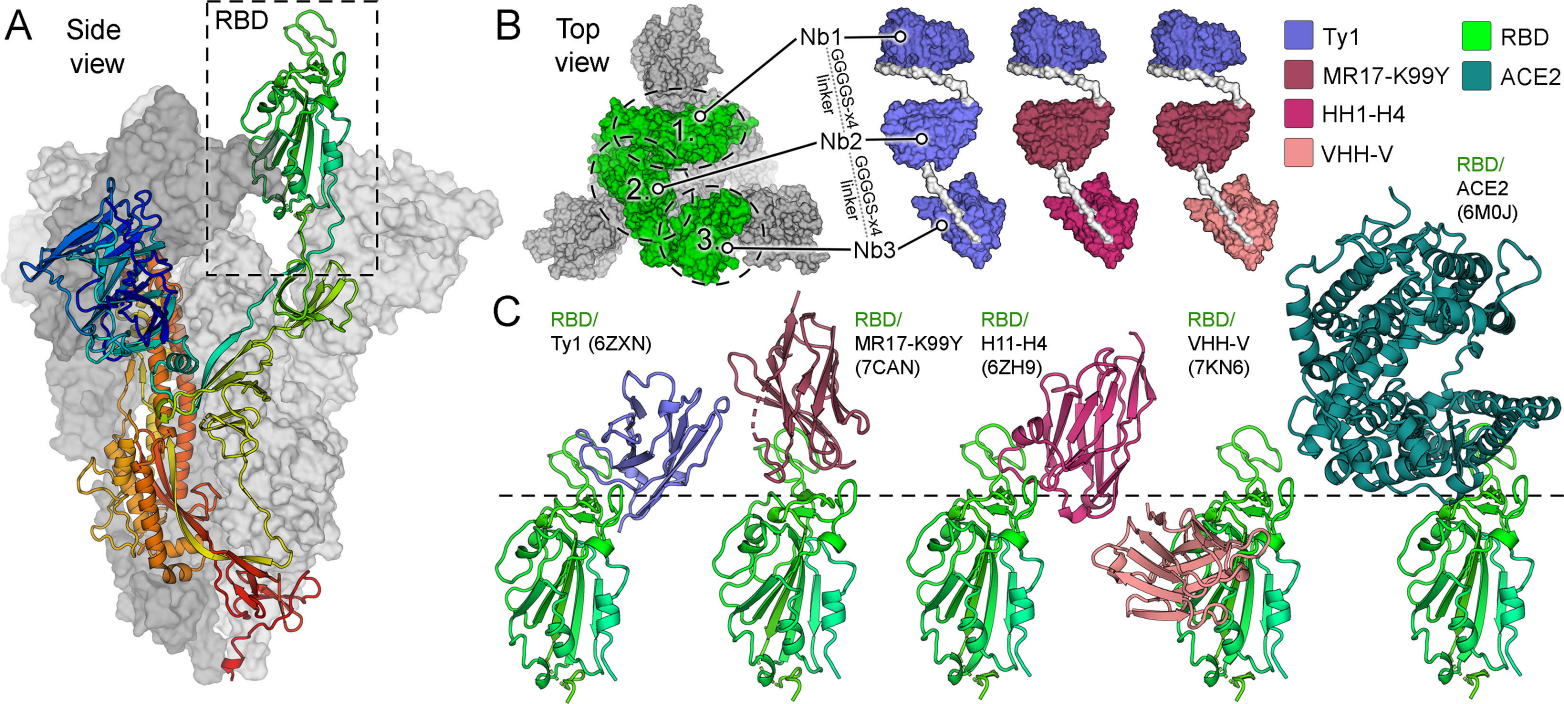
Structure-guided design of multimodular nanobodies targeting SARS-CoV-2 S (PDB 6ZXN). (**A**) Side view of the SARS-CoV-2 S-trimer (28). One trimer subunit, with a receptor-binding domain in the “up” conformation, is shown as a cartoon and colored as a rainbow ramped from blue (N-terminus) to red (C-terminus). The two other subunits are shown as surfaces in shades of gray. (**B**) Three distinct multimodular nanobodies were designed: (i) Tri-Ty1 with three repeats of Ty1 (28) module; (ii) Tri-TMH with Ty1, MR17-K99Y (30), and H11-H4 (29) modules; and (iii) Tri-TMV with Ty1, MR17-K99Y, and VHH-V (41) modules. The modules are connected by flexible GGGGSx4 linkers. (**C**) Binding sites of nanobody domains (Ty1, MR17-K99Y, H11-H4, and VHH-V) on the RBD. Comparison to the structure of RBD bound to the primary host-cell entry receptor of SARS-CoV-2, ACE2 (6, 48) demonstrates that three of the modules target the ACE2-binding site, while VHH-V targets an alternative neutralization epitope.

Inspired by the trimeric structure of the coronaviral spike (Fig. 1), we sought to develop an approach for targeting all RBDs simultaneously to enhance SARS-CoV-2 inhibition. Cryogenic electron microscopy (cryo-EM) studies have identified two SARS-CoV-2 RBD conformations, “up” and “down” (46, 47), where putative epitopes on neighboring RBDs are in proximity, within 40–74 Å from each other (Fig. S1). To develop a tripartite binder that is sterically accommodated within these tight constraints, we selected nanobodies, the smallest antibody-based protein inhibitors, as the base unit for multimodularization.

We designed three trimodular nanobodies (Fig. 1B) using the sequences of four previously published monomeric nanobodies, Ty1 (28), H11-H4 (29), MR17-K99Y (30), and VHH V (41), reported to neutralize wild-type SARS-CoV-2 with IC_50_ (half-maximal inhibitory concentration) values ranging from 38 to 142 nM (Table S1). These modules were selected based on two criteria: (i) distinct epitope and angle of binding to RBD (Fig. 1C; Table S1), and (ii) spatial proximity of the epitopes in the context of the SARS-CoV-2 spike, facilitating simultaneous binding of all modules (Fig. 1; Fig. S2).

Multimodular nanobodies were constructed by fusing three nanobodies together with flexible linkers of 20 amino acids (GGGGS×4) with the aim to improve binding avidity (26, 39, 49–51). Construct compositions were selected to test (i) the effect of triplicating a single module on SARS-CoV-2 neutralization, and (ii) whether the inclusion of variable modules helps mitigate neutralization escape (41). To this end, multimodular constructs Tri-Ty1 [comprising three Ty1 modules (28)], Tri-TMH, and Tri-TMV (Fig. 1B) were generated, with Tri-**TMH** and Tri-**TMV** comprising **T**y1 (28) and **M**R17-K99Y (30) modules, followed by either a **H**11-H4 (29) or VHH **V** (41) module, respectively.

### Multimodular nanobodies bind variant forms of the RBD

**Fig 2:**
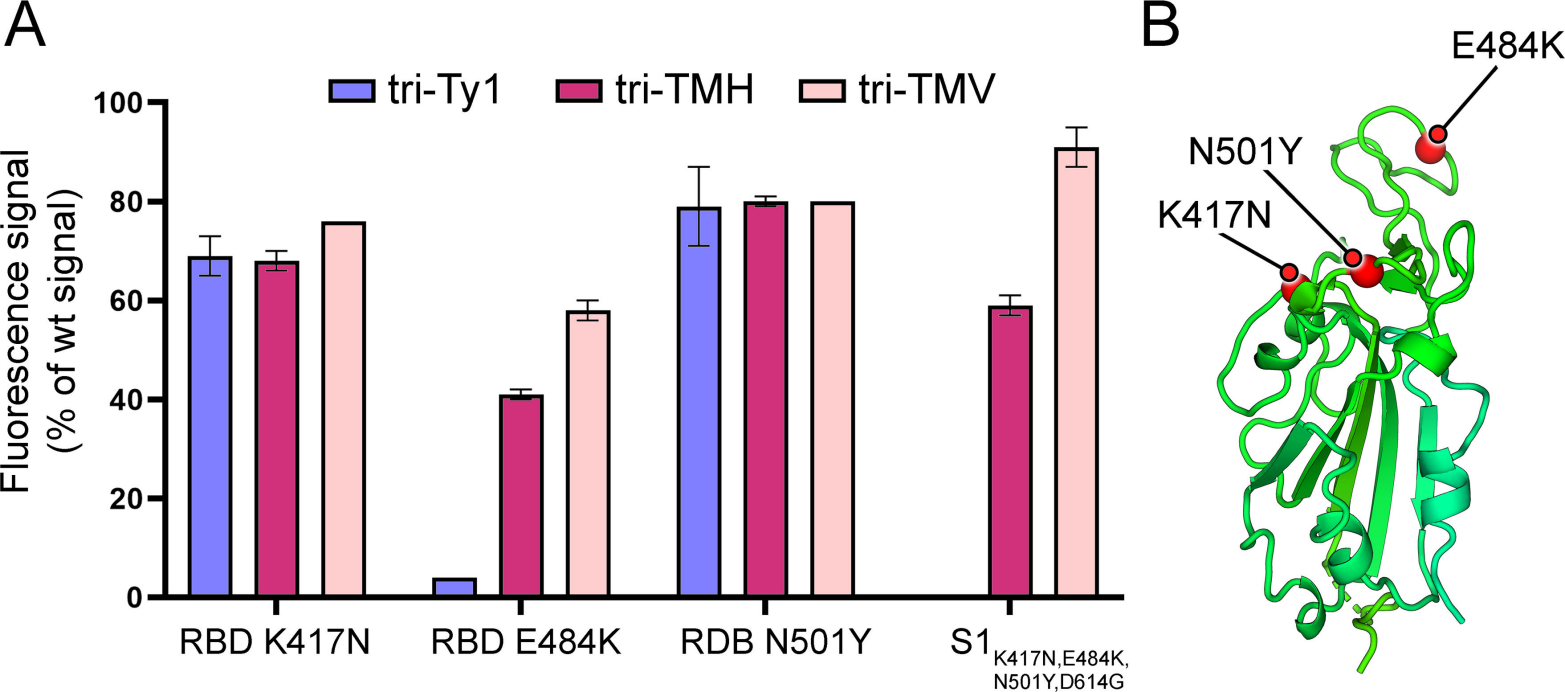
Relative binding strengths of trimeric nanobodies to RBD and Spike S1 domain variants on an antigen microarray. (**A**) Three multimodular nanobody constructs were tested for binding to RBD variants with amino acid changes K417N, E484K, or N501Y, and to spike the S1 domain with four amino acid changes (K417N, E484K, N501Y, and D614G). Fluorescence signals of Dylight 633-labeled multimodular nanobodies Tri-Ty1, Tri-TMH, and Tri-TMV bound to the different RBD and S1 variants are shown normalized relative to the signal from binding to wild-type RBD or S1 for each nanobody, respectively. Error bars represent the standard deviation of two replicate measurements. (**B**) Location of the amino acid changes within the SARS-CoV-2 RBD. Amino acid change N501Y is found in the Alpha and Beta variants (15, 16), and the Beta variant displays the additional changes K417N and E484K (16). Delta variant carries none of the three tested RBD amino acid changes, while Omicron displays N501Y, K417N, and an alternative change at residue 484, E484A.

Amino acid changes observed in SARS-CoV-2 VOCs have been linked to escape from antibody-mediated neutralization (15, 16, 52, 53) due to reduced affinity to epitopes with altered amino acids. To determine how RBD amino acid changes K417N, E484K, and N501Y impact multimodular nanobodies, we tested their binding to RBD and spike mutants in an antigen microarray. Nanobodies Tri-Ty1, Tri-TMH, and Tri-TMV were tested against (i) wild-type RBD, (ii–iv) three RBD variants that displayed either K417N, E484K, or N501Y amino acid change, (v) wild-type S1 subunit, and (vi) a S1 subunit displaying amino acid changes K417N, E484K, N501Y, and D614G. Tri-TMH and Tri-TMV each contain three distinct nanobody modules targeting a broad range of residues and, therefore, were expected to be less sensitive to amino acid changes than Tri-Ty1. The results support this hypothesis, showing that binding of Tri-Ty1 to RBD was strongly decreased (96% decrease) by the E484K amino acid change, which is found in the Beta VOC and linked to neutralization escape (12, 16, 52), while this effect was mitigated in Tri-TMH and Tri-TMV (~60% and 40% decrease, respectively; Fig. 2A). Amino acid changes K417N or N501Y did not affect binding to the same degree, resulting in 20%–30% decrease overall.

### Multimodular nanobodies potently neutralize SARS-CoV-2 wild-type and Alpha

**Fig 3:**
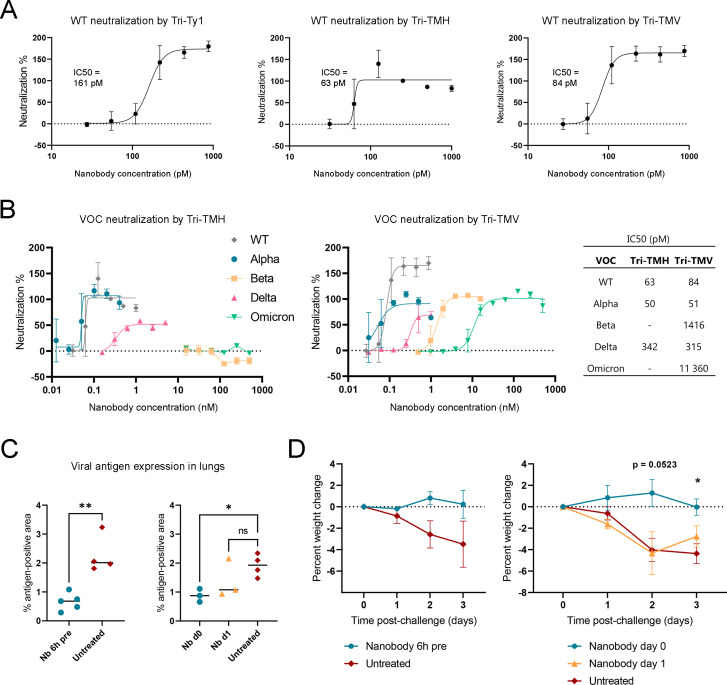
Multimodular nanobodies neutralize SARS-CoV-2 wild type, Alpha, and Delta variants at high potency and limit lung pathology in a hamster model. (**A**) Wild-type SARS-CoV-2 is neutralized by nanobodies Tri-Ty1, Tri-TMV, and Tri-TMH in a microneutralization assay. (**B**) Tri-TMH neutralizes the Alpha and Delta variants, but Beta and Omicron BA.1 escape neutralization. Tri-TMV neutralizes all variants tested, with lower potency for Beta and Omicron BA.1. Data shown are mean values from triplicate or quadruplicate experiments with error bars representing standard deviation. Data for Tri-TMH tested against Delta are from duplicate experiments. Curve-fitting and IC_50_ calculation were performed for the normalized neutralization data with GraphPad Prism using the nonlinear regression method. (**C**) Morphometric immunohistology assay was used to detect SARS-CoV-2 antigens in hamster lung tissue sections, and the data are shown as the percentage of lung tissue with viral antigen expression, with a line designating the median. Treatment groups received Tri-TMH nanobody intranasally 6 h prior to infection (Nb 6 h pre, *n* = 5), simultaneously to infection (Nb d0, *n* = 3), or 1 day after infection (Nb d1, *n* = 3). Unpaired *t*-test was used for the comparison of groups. *P ≤0.05 and **P ≤ 0.01. (**D**) Hamsters were weighed daily, and the relative weight change from the day of infection (day 0) is shown (mean ± SE). Two-way ANOVA with Dunnett’s multiple comparisons test or Šídák’s multiple comparisons test was used for the comparison of groups. *P ≤ 0.05.

We determined the neutralization potency of multimodular nanobodies against SARS-CoV-2 variants *in vitro*, using a plaque-reduction neutralization assay in VeroE6-TMPRSS2-H10 cells (Fig. 3). Multimodular nanobodies neutralized wild-type virus (50 pfu) at ultra-high potency, with IC_50_ values ranging from 161 pM for Tri-Ty1 to 84 pM for Tri-TMV and 63 pM for Tri-TMH, indicating up to 1,000-fold increase over constituent nanobodies (Table S1). Neutralization was also tested with virus administered at 30,000 pfu (1 MOI [multiplicity of infection]), where the relative efficacies of the nanobodies followed the same trends as in the 50 pfu assay, although at lower IC_50_-values ranging from 400 pM to 2 nM (Fig. S3).

To investigate the potency of multimodular nanobodies against SARS-CoV-2 variants, we tested Tri-TMH and Tri-TMV, the two most potent neutralizers of wild-type SARS-CoV-2, against the VOCs Alpha, Beta, Delta, and Omicron BA.1. Tri-TMH neutralizes SARS-CoV-2 Alpha variant effectively (IC_50_ = 50 pM) and Delta variant with lower potency still within picomolar range (IC_50_ = 342 pM). Beta and Omicron variants, however, escaped neutralization (Fig. 3B). This contrasts with the antigen microarray data (Fig. 2), where nanobodies with three distinct modules were found to retain most of their binding to RBD with key amino acid changes, which may be due to the sensitive binding array method detecting weak binding insufficient for neutralization at tested nanobody concentrations. Indeed, when significantly higher nanobody concentrations were used, Tri-TMV was found to neutralize all VOCs tested, including Beta (IC_50_ = 1.4 nM) and Omicron (IC_50_ = 11.4 nM) variants (Fig. 3B). Our findings underscore the importance of corroborating binding studies with assays using infectious viruses.

The inhibitory effect observed in neutralization assays against SARS-CoV-2 wild type was recapitulated in an animal model for SARS-CoV-2 infection. We tested the *in vivo* efficacy of the Tri-TMH nanobody, the most potent neutralizer identified by our *in vitro* neutralization assays, in 8-week-old Syrian golden hamsters. A 30 µg dose of nanobody was administered to six hamsters intranasally 6 h before inoculation with wild-type (Wuhan-Hu-1) SARS-CoV-2. The daily clinical monitoring revealed a slight drop in weight on day 1 in most animals, which was progressive in three of the four control hamsters but only observed in one treated hamster (Fig. S4) until day three post-infection, when the hamsters were euthanized and tissue samples collected. SARS-CoV-2 was detected and quantified in the lung tissue of the nanobody-treated group and an untreated control group via RT-qPCR of RdRp and E genes and via a morphometric analysis of the extent of viral antigen expression in the lungs, as shown by immunohistology. These analyses revealed a ~70% reduction of viral antigen-positive tissue area and a decrease in viral gene expression in nanobody-treated animals (Fig. 3C; Fig. S4). However, the viral antigen expression pattern did not differ between the groups (besides respiratory epithelial cells in the trachea and lower airways, some patches of alveoli with positive type I and II pneumocytes were observed), and the inflammatory response in the pulmonary parenchyma was generally mild, mainly represented by a multifocal increase in interstitial cellularity. Alternative treatment schedules were explored in a pilot experiment, where a 20 µg dose of nanobody was administered either immediately prior to the virus challenge or 1 day after the challenge. Administration immediately prior to infection mitigated weight loss and reduced the extent of viral antigen expression, in line with the results from the prophylactic approach (Fig. 3C and D; Fig. S4), while only very limited therapeutic effects were observed in the group that received nanobody 1 day after the challenge (Fig. 3C and D).

### Cryo-EM analysis reveals the conformational landscape of SARS-CoV-2 spike bound to a multimodular nanobody

While individual nanobody modules and their binding epitopes on the SARS-CoV-2 spike have been structurally characterized (28–37), these reconstructions do not account for the potential steric constraints imposed by linker-bound modules in multimodular nanobodies. The natural variation in SARS-CoV-2 spike conformation leads to the presence of distinct subpopulations in cryo-EM data, primarily fully closed (“all down”) and partially open (“one up”) states (4, 7, 54). While differential distributions of the two states have been reported, native spikes on the viral surface show 31% and 55% of fully closed and partially open conformations, respectively (54). Furthermore, certain neutralizing antibodies have been shown to disrupt the conformation of the spike due to steric incompatibility with the prefusion state (55, 56).

**Fig 4:**
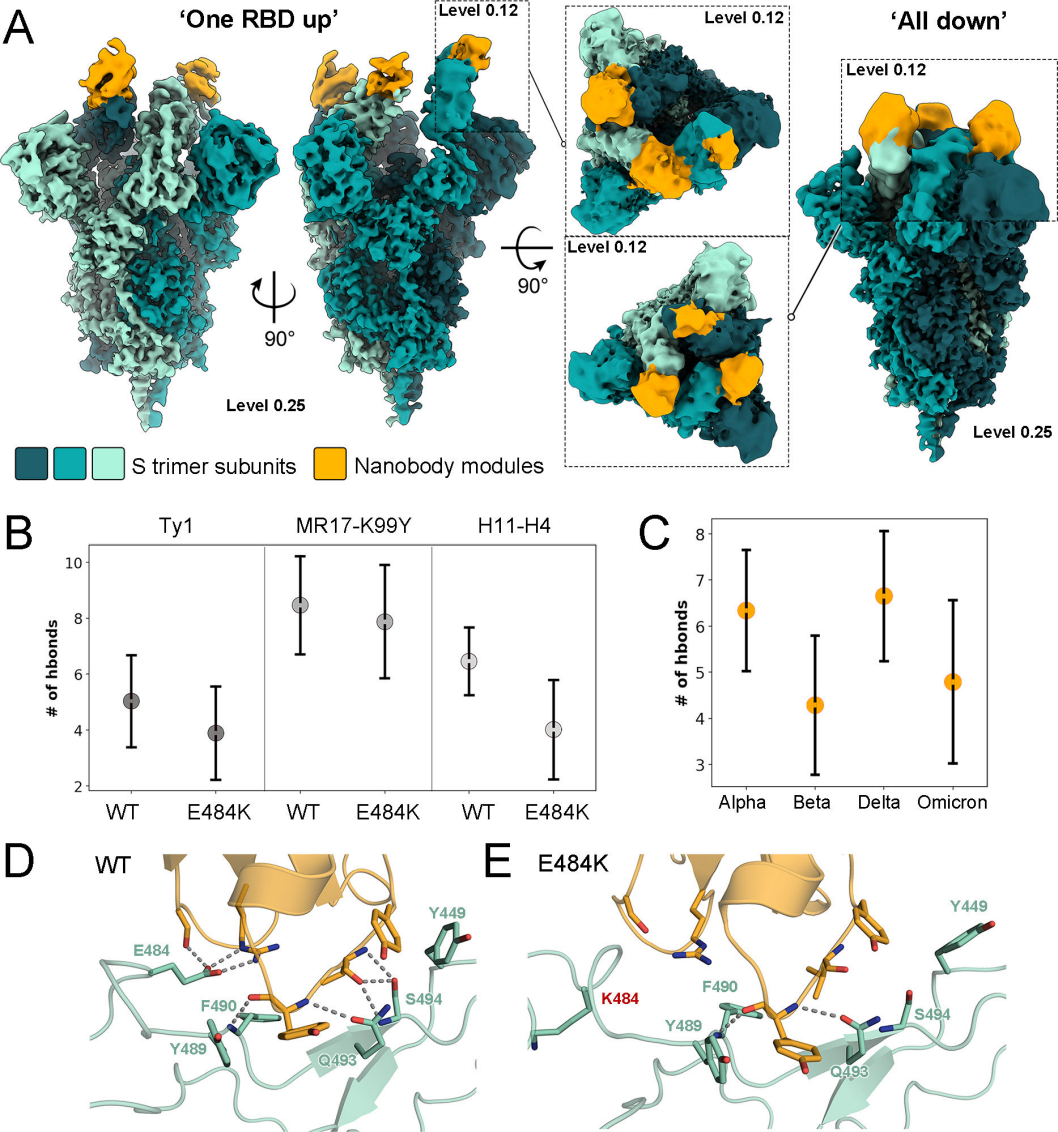
Insights into multimodular nanobody binding from cryo-EM analysis and molecular dynamics simulations. (**A**) Cryo-EM reconstructions of the S-trimer with multimodular nanobody Tri-TMH bound. The reconstructions show the typical spike protein conformations (“one RBD up” and “all RBDs down”). (**B**) Number of hydrogen bonds between RBD (wild type or with E484K amino acid change) and each nanobody module of Tri-TMH observed from MD simulation data. The dot shows the mean value based on all simulation replicas, and the line represents the standard deviation. (**C**) The number of hydrogen bonds between nanobody module H11-H4 and RBDs from SARS-CoV-2 VOCs, as predicted by MD simulations. A closer look at the binding interface after MD simulations shows that while the interface between nanobody module H11-H4 (orange) and wild-type RBD (green) remains stable (D), amino acid change E484K, highlighted in red, results in rearrangement that abolishes much of the hydrogen bonding network (E).

We set out to determine how nanobody Tri-TMH impacts the conformation of the spike. Our cryo-EM data show the spike retaining a prefusion conformation, with subpopulations presenting the closed and partially open states (Fig. 4; Fig. S5 to S7). Both states are fully bound with Tri-TMH (Fig. 4A), although due to the variable placement of the modules, local resolution (~3.3 Å for the S2 core; 5–7 Å at nanobody interfaces; Fig. S5) hinders the unambiguous identification of individual nanobody moieties. Focused refinement of each RBD region improved density features and allowed the identification of the three Tri-TMH modules bound to the RBDs in the partially open spike conformation, based on their distinct binding angles (Fig. S7). Our results indicate that the inclusion of multiple simultaneously binding modules, linked by (GGGGS)_4_ linkers, does not result in steric strain sufficient to disrupt the prefusion conformation, allowing the native distribution of RBD conformational states. We postulate that the increased potency of multimodular inhibitors is primarily due to enhanced avidity, not altered mechanistic properties.

### Molecular dynamics simulations indicate rearrangement of the nanobody-RBD interface as a result of amino acid changes present in VOC

Following the cryo-EM analysis, we sought to elucidate the molecular basis for neutralization evasion as observed for VOCs Beta and Omicron (Fig. 3B) via molecular dynamics (MD) simulations. Interestingly, many nanobodies, as well as antibodies derived from B cells of COVID-19 convalescent and vaccinated individuals, show a salt bridge between an antibody scaffold arginine (R52 in nanobodies) and residue E484 of the RBD (15, 16). As amino acid change E484K is linked to neutralization evasion by VOCs (16, 22, 57), we sought to determine how E484K impacts the interface of RBD and nanobody modules included in Tri-TMH. MD simulations of both WT RBD and E484K RBD were performed with the reported structures of the individual nanobody modules in complex with RBD (28–30).

For modules MR17-K99Y and H11-H4, the R52-E484 salt bridge was identified to be extremely stable throughout the WT simulations and in multiple independent simulation replicas (ca. 95% of total simulation time). However, when amino acid change E484K was simulated, the interaction to R52 was rapidly broken (within 1 ns). The dissociation of the salt bridge as a consequence of charge change from anionic glutamate to positively charged lysine contributed to higher instability of the E484K RBD, with the surrounding loop becoming more mobile (Fig. 4D and E; Fig. S8 and S9). In addition, the number of hydrogen bonds between RBD and the nanobody modules decreased in the E484K systems (Fig. 4B). Most of these disrupted hydrogen bonds were in the RBD-binding epitope (Fig. 4D and E). Additional simulations were performed for the H11-H4 module, with the full set of amino acid changes in the RBD for each VOC (Fig. 4C; Table S2). Here, the Alpha and Delta VOCs, where E484 is preserved, behaved similarly to the WT systems. However, both Beta and Omicron VOC, where E484 is substituted by lysine and alanine, respectively, showed higher instability (Fig. S8) and a decrease in hydrogen bonding interactions throughout the simulation (Fig. 4C). These data indicate that E484 is central for strong nanobody binding.

To further explore the role of this residue at nanobody−RBD interfaces, we calculated the impact of E484K amino acid change on the binding of the three modules of Tri-TMH, represented by the change in their binding enthalpies (ΔH) that were determined by Molecular Mechanics Poisson-Boltzmann Surface Area (MMPBSA) calculations on the MD trajectories (58, 59). We observed that upon introducing the E484K amino acid change, a weaker binding for both Ty1 and H11-H4 modules was observed, while the binding of module MR17-K99Y was less perturbed (Fig. S9). Our observations are in good agreement with previous computational studies, which showed similar trends for MR17-K99Y (22), indicating that the binding of this module remains similar for WT and E484K RBD, whereas the latter change indeed weakens the binding of both H11-H4 and Ty1 to the RBD (60, 61). Taken together, these data indicate that MR17-K99Y maintains its binding to the RBD with the E484K change, and thus may be responsible for the Tri-TMH binding to E484K RBD observed in the antigen microarray (Fig. 2). However, since we did not observe neutralization of viruses containing the E484K mutation with Tri-TMH (Fig. 3), we hypothesize that nanobody concentrations higher than 500 nM would be required to achieve effective neutralization.

### Nanobodies fused to split nanoluciferase fragments detect SARS-CoV-2 spike at picomolar concentrations

Here, we applied nanobody engineering in the development of a SARS-CoV-2 antigen detection assay. The modular nature of nanobodies makes them amenable to fusion with signal molecules, and their small size allows the targeting of proximal epitopes, such as those presented by the three subunits of the SARS-CoV-2 spike. These properties align with the principle of protein-fragment complementation, where the activity of a split signal molecule is restored once the split fragments are brought into proximity by the interaction of proteins fused to the fragments (62). Nanobodies shown in Fig. 1 target epitopes in the RBD, and we hypothesized that, when fused to split signal molecule fragments, their binding to three spike subunits can reconstitute the signal molecule, allowing sensitive detection in a single step.

**Fig 5:**
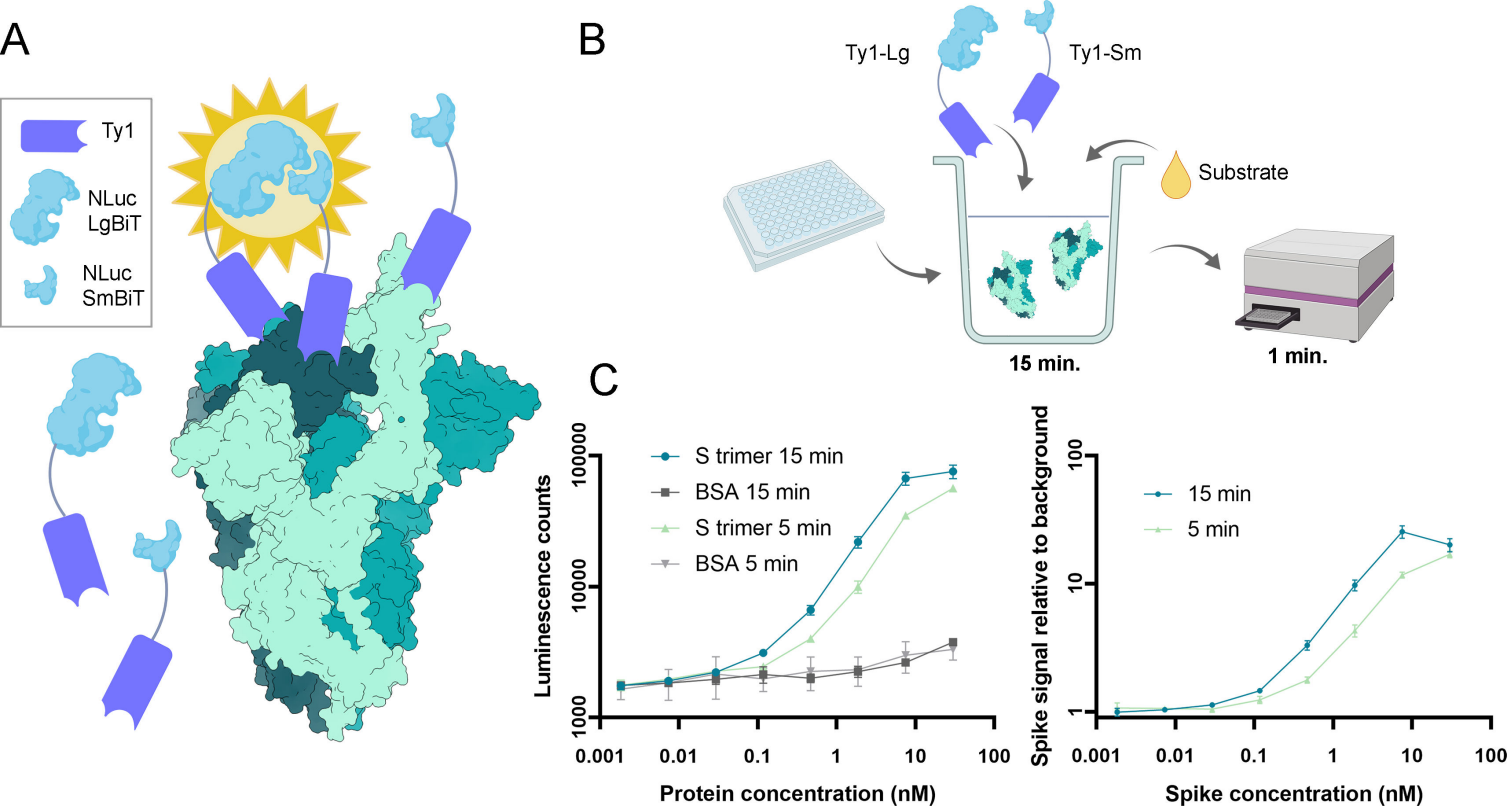
SARS-CoV-2 spike protein detection by a nanobody-based rapid antigen assay. (**A**) Principle of the assay. Split nanoluciferase fragments are fused to Ty1 nanobody (28) with GGGGS_4_ linkers. The enzymatic activity of luciferase is restored upon nanobody binding to adjacent RBDs. (**B**) Reaction setup. The nanobody fusions are added to a dilution series of SARS-CoV-2 S-containing samples on a 96-well plate, and the mixture is incubated for 15 min. Then, the substrate is added, and the luminescence reads are recorded from each well. (**C**) The signal induced by spike protein can be distinguished from background down to trimeric spike protein concentration of 200 pM. Data shown are mean values from a triplicate experiment with error bars representing standard deviation. Figure created with BioRender.com.

We selected the split version of NanoLuc, an engineered 19-kDa luciferase with enhanced stability and brightness (43, 44), as the signal molecule for the assay. RBD-binding nanobody Ty1 (28) was fused with fragments of NanoLuc, titled SmBit or LgBit (42, 44), using flexible (GGGGS)_5_ linkers to connect nanobodies with signal molecule fragments (Fig. 5). We performed proof-of-principle experiments to gauge the ability of the nanobody-nanoluciferase fusions to detect SARS-CoV-2 spike in solution. Ten nanomolar concentrations of Ty1-LgBiT and Ty1-SmBiT were mixed with a dilution series of either SARS-CoV-2 spike or a negative control protein (bovine serum albumin, BSA) and incubated for 15 min before substrate addition and luminescence measurement. The luminescence resulting from the addition of SARS-CoV-2 spike in the reaction can be distinguished from background at a concentration as low as 200 pM (Fig. 5). In addition, we tested the assay with UV-inactivated wild-type SARS-CoV-2 and human coronaviruses 229E and NL63. SARS-CoV-2 was detected, albeit at a lower limit of detection (~1 × 10^8^ FFU/mL) than the spike protein, presumably due to the UV inactivation and the freeze-thaw cycle required to run the assay outside of BSL-3. The other human coronaviruses were not detected by the assay, indicating specific detection of SARS-CoV-2 (Fig. S10). These proof-of-principle experiments demonstrate the potential of nanobodies in the development of diagnostics.

## DISCUSSION

In this study, we engineered potent SARS-CoV-2 neutralizers and detection tools through fusions of spike-targeted nanobody modules. Our multimodular nanobodies had IC_50_ values in the 50–160 pM range (Fig. 3A and B), indicating a notable increase (up to 1,000-fold) relative to the reported IC_50_ values of the single constituent nanobodies. This observation is in line with recent works reporting nanobody multimerization increasing neutralization potency (38, 41, 63, 64). While nanobody treatment did not eliminate SARS-CoV-2 infection in the animal model, prophylactic administration of Tri-TMH in the nasal cavity limited viral replication in the lungs and alleviated the clinical signs, as reflected by the prevention of weight loss (Fig. 3). As multivalent small protein inhibitors comprise an emerging category of antivirals, not many studies are available where similar animal models were used, but we note that our results are in line with recent reports where nanobody treatment limited weight loss and viral gene expression in the lungs following intranasal delivery of homotrimeric (27, 65) or divalent (30) nanobodies to Syrian golden hamsters infected with SARS-CoV-2.

In neutralization assays with VOCs, Alpha and Delta were neutralized, while Beta and Omicron either escaped neutralization completely or required considerably higher nanobody concentrations to be neutralized. The finding that Tri-TMV neutralizes Beta and Omicron, while Tri-TMH does not, can be attributed to the VHH V nanobody module that targets a distinct site in the RBD (Fig. 1), which is more conserved than the ACE2-binding interface (41, 66). Our MD simulations indicate that the E484K amino acid change results in a loss of salt bridge with conserved R52 and conformational rearrangement in the receptor-binding domain (Fig. 4), causing disruption of the nanobody-binding interface and leading to neutralization escape in Beta and Omicron variants. MD simulation data also revealed interaction trends (Fig. 4, panels D and E) at the antigen interface, which correlate with the experimentally observed neutralization (Fig. 3). We postulate that this approach, focused on simulating hydrogen bonding networks and root-mean-square deviation (RMSD) trends at the antigen interface, may be applied to qualitatively predict the potential for escape from specific neutralizers.

Drawing from protein-fragment complementation assays, we were inspired to combine nanobody modules with signal molecules to develop new diagnostic tools. The assay was successful at detecting low concentrations (200 pM) of SARS-CoV-2 spike protein, in line with the limits of detection previously reported for assays detecting the spike antigen, ranging from ~4 to 500 pM (67–69). However, when testing our assay with SARS-CoV-2 virions, the limit of detection was poorer than in commercial antigen tests targeting SARS-CoV-2 nucleoprotein (40, 70). A potential strategy to optimize the assay for improved signal would be to target the viral nucleoprotein, which is more abundant in the virions than the spike, and is commonly used as an analyte in antigen tests (40, 70). As it stands, our assay specifically detects the SARS-CoV-2 virus with no cross-reactivity toward the other coronaviruses tested, providing valuable proof-of-concept data for the use of nanobodies and protein fragment complementation in virus detection. In contrast to monoclonal antibodies commonly used in diagnostics, engineered nanobodies have multiple attractive properties, including cheap and scalable production that does not require resource-intensive mammalian cell culture (49). Nanobodies can also be discovered from synthetic libraries, allowing the transition away from conventional animal immunization prevalent in antibody generation (71). We envision that a combination of nanobody modules with split signal molecules presents a powerful platform for the development of single-step detection assays for emerging pathogens.

We are faced with increasing rates of viral emergence. Nanobodies, being inexpensive and readily modified, show promise as both inhibitor candidates and diagnostic tools. Our work showcases the potential of nanobodies engineered to target viral pathogens to improve preparedness for outbreaks of emerging infectious diseases.

## MATERIALS AND METHODS

### Production and purification of multimodular and luciferase-fused nanobodies

Synthetic genes encoding multimodular nanobodies [individual modules reported in references (28–30, 41)] and nanobody fusions with split nanoluciferase (44, 45) were ordered cloned in expression vector pET100/D-TOPO (GeneArt, Thermo Fisher Scientific). Nanobodies were expressed in *Escherichia coli* Rosetta-gami 2 (DE3) cells (Novagen) in autoinduction media and purified through consecutive nickel affinity chromatography and size-exclusion chromatography (see Supplementary Methods).

### Production and purification of recombinant SARS-CoV-2 S protein

SARS-CoV-2 spike was expressed from a synthetic cDNA template (GeneArt, Thermo Fisher Scientific) encoding the S protein ectodomain residues 14–1,208 from the Wuhan-Hu-1 strain (NCBI reference sequence: YP_009724390.1) stabilized in the prefusion state (4, 7) with proline substitutions at residues 986 and 987, an abrogated furin S1/S2 cleavage site with a “GSAS” substitution at residues 682–685, and a C-terminal T4 fibritin trimerization motif. In our construct, the trimerization motif was followed by an HRV3C protease cleavage site, SpyTag003 (72), and 8xHisTag. The gene was cloned into the mammalian expression vector pHLsec and transfected into Expi293F (Thermo Fisher Scientific) suspension cells for expression. The protein was purified through nickel affinity chromatography (see Supplementary Methods for further details).

### Cryo-EM grid preparation, data acquisition, and data processing

A 3 µL aliquot of a pure, prefusion SARS-CoV-2 S-trimer (0.3 mg/mL) mixed with Tri-TMH (0.05 mg/mL) was applied on Quantifoil 1.2/1.3 grids (1.2 µm hole diameter, 200 mesh copper) that had been glow discharged in Plasma Cleaner PDC-002-CE (Harrick Plasma) for 30 s. The grids were blotted for 6 s and plunged into liquid ethane using a vitrification apparatus (Vitrobot, Thermo Fisher Scientific).

Data were collected on a Titan Krios transmission electron microscope (Thermo Fisher Scientific) equipped with a Gatan K2 direct electron detector. EPU v 2.11.0 software was used to acquire micrographs, and images were collected with a dose of 1.38 e⁻/Å² per image (Table S3).

Data were processed in cryoSPARC (73). Movie frames were aligned and averaged to correct for beam-induced motion. Contrast transfer function parameters were estimated using CTFFIND4 (74). An initial set of particles, picked with the blob-picker, was classified, and the particles in good 2D classes were used to train the Topaz particle picker (75, 76). A total of 91,601 particles were selected after cleaning the picked set with 2D classification. An initial volume with C3 symmetry was calculated *ab initio*.

### Refinement of cryo-EM maps

Following the generation of an *ab initio* volume with C3 symmetry, a consensus map of the S trimer, with C3 symmetry applied, was resolved to 2.66 Å resolution. Visual inspection showed that the RBD region was poorly defined in the consensus map. 3D variance analysis of symmetry-expanded particles was run using a spherical mask defining the RBD region, six principal modes (i.e. eigenvectors of the 3D covariance), and eight classes (or clusters). Particles in each class were subjected to local asymmetric refinement (standard deviation over the prior rotations and shifts were 5° and 5 Å, respectively, centered at the box center). This local refinement prevented symmetry-expanded particles from rotating over their symmetry copy. The resolution of the maps was estimated based on the gold-standard Fourier shell correlation criterion of 0.143 (77), and the final maps were filtered to local resolution. CryoEM data collection and processing statistics are presented in Table S3. Additionally, to enhance features in regions of interest for model fitting, each RBD region density from both “one-up” and “all-down” conformation reconstructions was further refined by local, focused refinement using particles where the bulk of the S-trimer density had been subtracted.

### Model fitting into cryo-EM maps

To fit molecular models of the S trimer (PDB: 7A29) and nanobody-RBD complexes (PDB: 6ZHD, 6ZXN, and 7CAN) in the cryo-EM density map, “fitmap” and “matchmaker” functions were used in USCF Chimera (78). The “fitmap” function was used to simulate a density for each PDB model to the global resolution of each map (3.48 Å for the all-down map and 3.28 Å for the one-up map). The S trimer model was placed first, yielding placements with map-to-map correlation scores of 0.7269 for the one-up map and 0.7367 for the all-down map. The “matchmaker” function in UCSF Chimera was used to match models of RBDs with the RBDs of the full spike model. To account for the mobility of the RBDs, the individual RBD models were further fitted into the spike reconstructions by the “fitmap” function (correlation scores for RBD models: one-up map 0.8064, 0.7843, and 0.7824; all-down map 0.7937, 0.7799, and 0.8017). Finally, RBD-Nb models (PDB: 6ZHD, 6ZXN, and 7CAN) were superposed to the fitted RBDs with the matchmaker function for the all-down map. For the partially open map, with one RBD in the up conformation, localized refinement enhanced density features and allowed the identification of nanobody modules bound at each location by using the “fitmap” function in ChimeraX (global search with 500 initial placements and starting with models outside of density, maps for nanobody-RBD complexes were simulated at 6 Å resolution). The function found the top placement for each nanobody-RBD complex at a distinct site, yielding map-to-map cross-correlation scores of 0.86 for Ty1—RBD (from 6ZXN), 0.82 for MR17-K99Y—RBD (7CAN), and 0.87 for H11-H4—RBD.

### Proof-of-concept tests for the detection assay with Split NanoLuc-nanobody fusions

A triplicate dilution series of purified recombinant SARS-CoV-2 spike in Tris buffer (10 mM Tris-HCl pH 7.5 and 150 mM NaCl) was made on an opaque white 96-well plate. In each well, purified Ty1-LgBiT and Ty1-SmBiT were added at 10 nM final concentration. The plate was incubated for 15 min at room temperature, after which nanoluciferase substrate coelenterazine H was added to 200 nM concentration. Luminescence readings were measured with a Perkin-Elmer EnSpire multimode plate reader using the “Luminescence” program. To determine the signal-to-noise ratio in the luminescence reaction, the assay was performed on an equimolar dilution series of BSA. To calculate the final luminescence measurement while taking noise into account, the average readings of each triplicate sample were divided by corresponding averages from the BSA controls. The assay was further validated with samples of SARS-CoV-2 WT and human coronaviruses hCoV-NL63 and hCoV-229E, using the same protocol. Before transferring viruses to BSL-1 conditions, the samples were UV inactivated in 1 mL aliquots with a dose of 5,000 × 100 mJ (UV Crosslinker, CL-1000, Jena Analytik).

### Antigen array

Nanobodies were labeled with DyLight 633 in PBS supplemented with 50 mM sodium borate (pH 8.5) at 1 mg/mL protein concentration and 50 µM Dylight 633 NHS ester (Thermo Fisher Scientific) at room temperature for 2 h, followed by removal of unreacted dye with Zeba Spin 7K MWCO desalting columns (Thermo Scientific).

Wild-type and variant SARS-CoV-2 RBD and spike S1 domains were biotinylated and arrayed as duplicate spots (0.1 ng per spot) in the wells of streptavidin-coated microtitration plates using a piezoelectric non-contact microarray dispenser (Nano-Plotter, GeSiM, Germany). The antigens were purchased from the following sources: RBD wt (aa 319–541 of the S protein) and S1 wt (aa 14–681 of the S protein) from Medix Biochemica; RBD single mutants K417N, E484K and N501Y and S1(K417N, E484K, N501Y, D614G) quadruple mutant from SinoBiological.

The antigen arrays in microplate wells were blocked with 50 µL of Assay buffer [Tris-buffered saline (TBS), pH 8.0 + 0.05% Tween 20] per well for 30 min at RT, followed by three washes with washing buffer (TBS containing 0.05% Tween 20). DyLight 633-labeled nanobody solutions (1 µg/mL in Assay buffer) were added 50 µL/well, incubated in a plate shaker at 600 rpm, RT for 1 h, followed by three washes with washing buffer. Residual liquid droplets were removed by centrifuging the plate upside down on a paper towel in a plate adapter (453 *g*, 1 min), after which the plate was left to dry for 15 min in a 37°C room. The Dylight 633-labeled nanobodies bound to the arrayed antigens were detected by fluorescence scanning through the clear bottom of the microplate with a Tecan LS400 confocal laser scanner, using a 633 nm laser for excitation and a 670/25 emission filter.

The fluorescence scan images were analyzed with Array-Pro Analyzer software (Media Cybernetics), and the raw spot signal data were exported to Microsoft Excel for further calculations. Net signals were obtained by subtracting the well background from the raw spot signals (average pixel intensity in the spot area), after which the spot signals were normalized to the wild-type antigen spot signals in the same well: single RBD mutant signals were expressed as a percentage of the RBD wt signal, whereas the quadruple S1 mutant signal was expressed as a percentage of the S1 wt signal.

### Neutralization assays

VeroE6-TMPRSS2-H10 cells (67) were seeded to 96-well plates (white-sided, optically clear bottom, PerkinElmer) in density 30,000 cells/well 24 h before the assay. The nanobodies were diluted in a virus growth medium (VGM) containing MEM (Sigma, 2279), 2% FBS, L-glutamine, and 1× penicillin-streptomycin. Diluted nanobodies were mixed with 50 pfu (Fig. 3) or 30,000 pfu (Fig. S3) of virus and incubated for 0.5–1 h at 37°C and 5% CO_2_. Thereafter, cells were treated with a mixture of nanobodies and virus. Experiments were performed in triplicate or quadruplicate, a virus dilution in VGM without nanobodies was used as a negative control, and non-infected cells (MOCK) were used as a control for cell viability. After 5 days of incubation, the medium was removed, and cells were treated with CellTiter-Glo 2.0 cell viability assay reagent (Promega, G9243) for 20 min at RT. Then, the cellular ATP was measured via the detection of a luminescent signal using the Hidex Sense microplate reader (Hidex Oy, Finland). Viability of the MOCK-infected cells was considered as 100%. The neutralization efficacy percentage for each measurement was calculated considering MOCK-infected cells as 100% neutralization and untreated, virus-infected cells as 0% neutralization. Curve fitting and IC_50_ calculation were performed for the normalized neutralization data with GraphPad Prism version 9.5.1 for Windows, using the nonlinear regression method (“Absolute IC50, X is concentration” function), with baseline constraint set to zero.

### Animal experiments

A total of 20 Syrian Golden hamsters (Scanbur, Karl Sloanestran, Denmark) were moved to the University of Helsinki biosafety level-3 facility and allowed to acclimatize to individually ventilated biocontainment cages (ISOcage; Scanbur, Karl Sloanestran, Denmark) for 7 days with *ad libitum* water and food (rodent pellets) prior to infection.

For the main experiment, six 8-week-old male and female Syrian Golden hamsters received 30 µg of nanobody, in 0.61 mg/mL concentration, 6 h prior to intranasal inoculation with 5 × 10^4^ SARS-CoV-2 (wt/D614G strain). The control group received an equal volume of PBS (*n* = 4).

Euthanasia was performed under terminal isoflurane anesthesia with cervical dislocation, followed by dissection immediately after death. Samples were collected from the lungs for RT-qPCR (see Supplementary Methods), and the remaining lung tissue, including trachea, heart, esophagus, and bronchial lymph nodes, was immersed in 10% buffered formalin. After 48 h, the tissue was stored in 70% ethanol until processing for histological and immunohistological examination.

### Histological, immunohistological, and morphometrical analyses

Three to five cross-sections were prepared from the fixed lung tissue and routinely paraffin wax embedded. Consecutive sections (3–5 µm) were prepared and stained with hematoxylin-eosin for histological examination or subjected to immunohistological staining for SARS-CoV-2 antigen expression, using a previously published staining protocol (65). For immunohistology, the horseradish peroxidase method was applied. Rabbit anti-SARS-CoV nucleocapsid protein (Rockland, 200-402-A50) served as the primary antibody, and DAB (EnVision FLEX DAB + Chromogen in Substrate buffer; Agilent) for visualization of antibody binding. All incubations took place in an autostainer (Dako). Sections were subsequently counterstained with hematoxylin.

For morphometric analysis, the immunostained sections were scanned (NanoZoomer-XR C12000; Hamamatsu, Hamamatsu City, Japan) and analyzed using the software program Visiopharm (Visiopharm 2020.08.1.8403; Visiopharm, Hoersholm, Denmark) to quantify the area of viral antigen expression in relation to the total area (area occupied by lung parenchyma) in the sections. This was used to compare the extent of viral antigen expression in the lungs between untreated and treated animals. A first app was applied that outlined the entire lung tissue as region-of-interest (ROI) (total area). For this, a Decision Forest method was used, and the software was trained to detect the lung tissue section (total area). Once the lung section was outlined as ROI, the lumen of large bronchi and vessels was manually excluded from the ROI. Subsequently, a second app with Decision Forest method was trained to detect viral antigen expression (as brown DAB precipitate) within the ROI.

### Molecular dynamics simulations of nanobody-RBD interface

MD simulations were performed on the model systems constructed using the cryo-EM structure of nanobody monomers and RBD (PDB: 6ZXN, 7CAN, and 6ZBP). During model system construction, long glycine linkers were removed. VMD psfgen tool (79) was employed to add missing hydrogen atoms, make point mutations, and solvate the systems with TIP3P water and 0.1 M NaCl to give a total system size of ca. 150,000 atoms. The force field for all components of the system was CHARMM36 (80). GROMACS v20.3 (81) was used for all equilibration and production simulations. First, the initial systems were minimized with restraints on heavy protein atoms (20,000 kJ mol^−1^ nm^−2^). Following convergence, a 100 ps NVT equilibration was performed with the same restraints, followed by a 10 ns NPT with the restraints only on the protein backbone atoms. All restraints were removed for the production runs, which employed the LINCS algorithm (82) to achieve a 2 fs timestep and used the Nosé-Hoover thermostat (83, 84) to maintain 310 K temperature and the Parrinello-Rahman barostat (85) to maintain 1 atm pressure. The electrostatic interactions were controlled by the particle mesh Ewald method (86) with a 12 Å cutoff, while the van der Waals cutoff was also 12 Å, with a switching distance of 10 Å. Multiple independent simulation replicas were performed and trajectories were visualized and analyzed using VMD (79) and Pymol (The PyMOL Molecular Graphics System, version 1.2r3pre, Schrödinger, LLC). Table S2 lists system setups and their respective lengths.

To calculate the binding affinity of each Tri-TMH module and RBD, the MMPBSA calculations were performed (58, 59). The MD trajectories of each replica were combined after excluding the first 100 ns of data to calculate the mean binding energy for each system (3 × 4,000 frames for each system). Note that we report here ΔH values, instead of ΔG, due to large uncertainties in calculating the entropic term.

### Molecular graphics

Molecular graphics images were generated using PyMOL (The PyMOL Molecular Graphics System, version 2.4.0.0, Schrödinger, LLC), UCSF Chimera (78), and UCSF ChimeraX (87).

## Data Availability

Cryo-EM reconstructions of the nanobody-bound SARS-CoV-2 spike in the one-up and all-down states have been deposited at EMDB under the accession codes EMD-19064 and EMD-19068.

## SUPPLEMENTAL MATERIAL

Supplemental material spectrum.04199-22-s0001.pdf Figures S1 to S10, Tables S1 to S3, and supplemental methods.

ASM does not own the copyrights to Supplemental Material that may be linked to, or accessed through, an article. The authors have granted ASM a non-exclusive, world-wide license to publish the Supplemental Material files. Please contact the corresponding author directly for reuse.
