# Supplementary material for: Nanobody engineering for SARS-CoV-2 neutralization and detection
Source: Microbiol Spectr. 2024 Feb 16;12(4):e04199-22. doi: 10.1128/spectrum.04199-22 (PMC10986514; doi:10.1128/spectrum.04199-22)
Supplement: Supplemental material — Figures S1 to S10, Tables S1 to S3, and supplemental methods. [file spectrum.04199-22-s0001.pdf]

Supporting information

Nanobody engineering for SARS-CoV-2 neutralization and detection

Liina Hannula, Suvi Kuivanen, Jonathan Lasham, Ravi Kant, Lauri Kareinen, Mariia Bogacheva, Tomas Strandin, Tarja Sironen, Jussi Hepojoki, Vivek Sharma, Petri Saviranta, Anja Kipar, Olli Vapalahti, Juha T. Huiskonen, and Ilona Rissanen

Items:

Figure S1. Distances between key residues in SARS-CoV-2 spike

Figure S2. Modelling of spike-bound nanobody modules connected by $(GGGGS)_4$ linkers

Figure S3. Neutralization of SARS-CoV-2 WT at 1 MOI by multimodular nanobodies

Figure S4. RT-qPCR of hamster lung samples and individual hamster weights

Figure S5. Resolution estimates of cryo-EM reconstructions

Figure S6. Nanobody-bound spike cryo-EM maps rendered at different contour levels

Figure S7. Fit of spike and nanobody PDB models in the cryo-EM maps

Figure S8. RMSD of protein Cα atoms over MD simulation and distance between charged residues (E/K and R) in MD simulations

Figure S9. Binding enthalpy (ΔH) of each tri-TMH module and SARS-CoV-2 RBD.

Figure S10. Detection of UV-inactivated SARS-CoV-2 using nanobodies fused with split nanoluciferase

Table S1. Reported binding and neutralization properties of nanobody modules

Table S2. Parameters for the molecular dynamics simulations

Table S3. Cryo-EM data collection and processing statistics

Supplementary Methods

Supplementary References

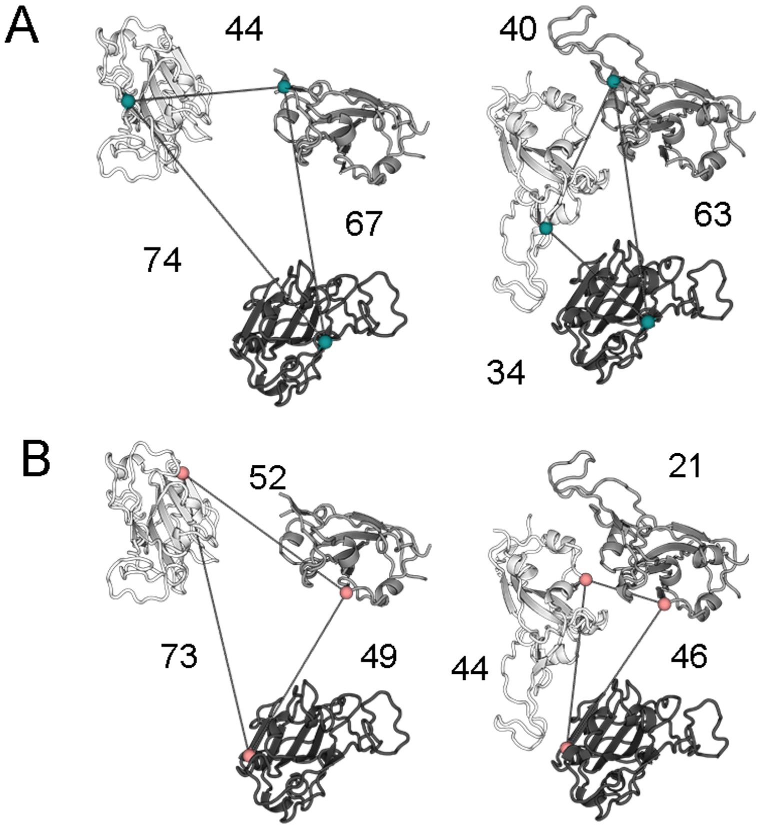

Figure S1. Distances (Å) between key residues in the receptor-binding domains in the different conformations of the SARS-CoV-2 spike illustrate the distance bridged by nanobody modules connected by 20-AA linkers. **A**) Distances between the central residues (493) of the ACE2 binding sites in the 3 RBDs, with spike in the 2-up conformation (left) or 1-up conformation (right). **B**) Distances between the central residues (375) of the VHH V nanobody epitopes in the 3 RBDs, with spike in the 2-up conformation (left) or the 1-up conformation (right).

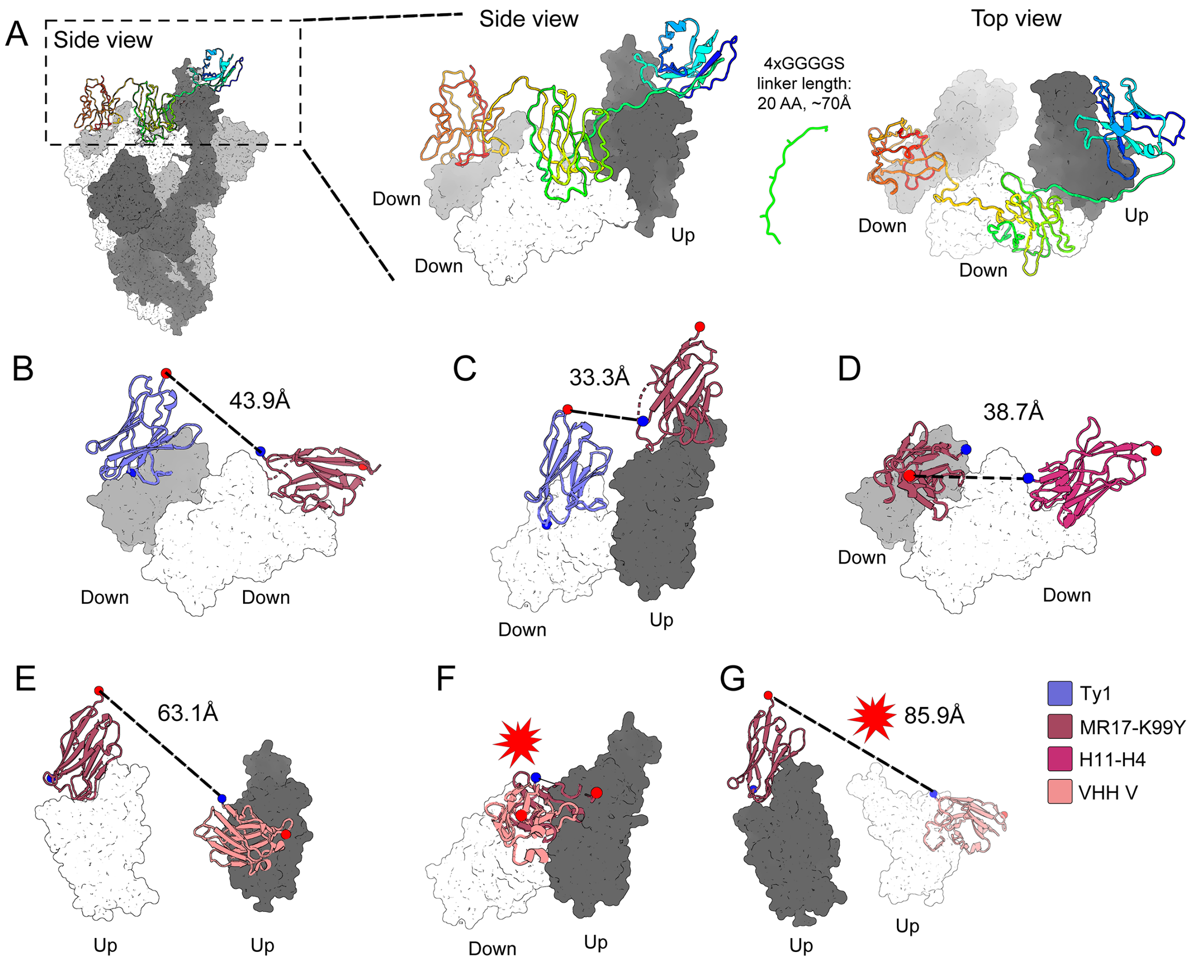

Figure S2. Distances between nanobody modules bound to different RBD conformations. The figure illustrates the length of the $(GGGGS)_4$ linker connecting the nanobody modules, and the distances the $(GGGGS)_4$ linkers must cover between the C-terminus of one nanobody module and the N-terminus of the next. **A**) A model of a multimodular nanobody bound to SARS-CoV-2 spike, with modules connected by $(GGGGS)_4$ linkers. Linker residues were built in Coot (1) between nanobodies in a model of Ty1 binding the spike protein (PDB 6ZXN), resulting in a model of the spike-bound multimodular nanobody tri-Ty1. The selected 20-residue linker is sufficiently long to span a distance of approximately 70Å between nanobody modules. **B**)-**E**) Distances (Å) between the nanobody C termini (red spheres) and N termini (blue spheres) are shown. There are more than 20 possible module pair/RBD conformation combinations, and these panels show examples of nanobody modules binding adjacent RBDs in either up or down conformations. Linker length accommodates binding to RBD pairs in the prevalent down-down and down-up conformations, and also allows binding to some of the rarer up-up RBD conformations. Panels **F-G**) shows a nanobody-spike conformation that are unlikely to be allowed, due to a steric clash (panel F), or due to a distance between nanobodies that the $(GGGGS)_4$ linker may not bridge (panel H). These models demonstrate that the linker length is permits multivalent binding to most RBD conformations. PDB models used for the analysis were 6ZXN (spike in 1-up conformation), 7DX8 (spike in 2-up conformation), 6ZXN (Ty1 in complex with Spike), 7CAN (MR17-K99Y in complex with RBD), 6XHD (H11-H4 in complex with Spike) and 7KN6 (VHH V in complex with RBD).

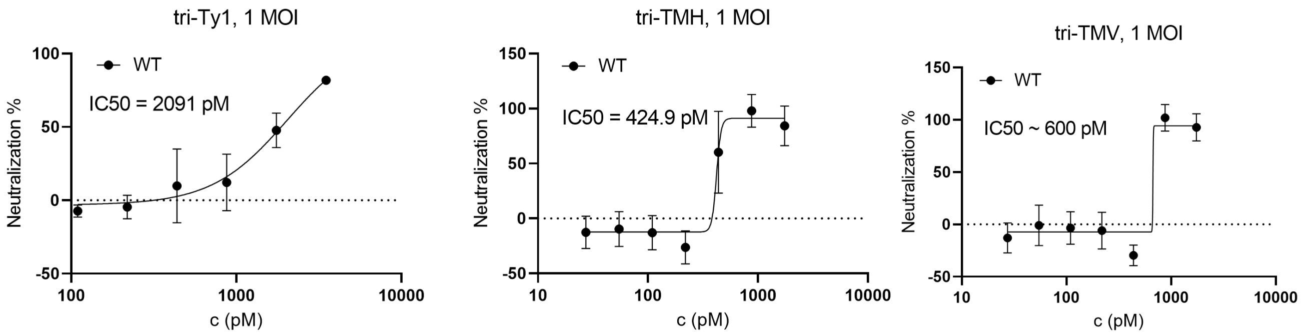

Figure S3. Parallel neutralization experiments were performed with 30 000 pfu (1 MOI) of virus instead of 50 pfu (Main Figure 3). Nanobodies tri-Ty1, tri-TMH, and tri-TMV show neutralization at these higher virus titers, but the calculated IC50 values are decreased. With higher amounts of virus used for infection, tri-TMH remains the most effective neutralizer, based on the calculated IC50 value.

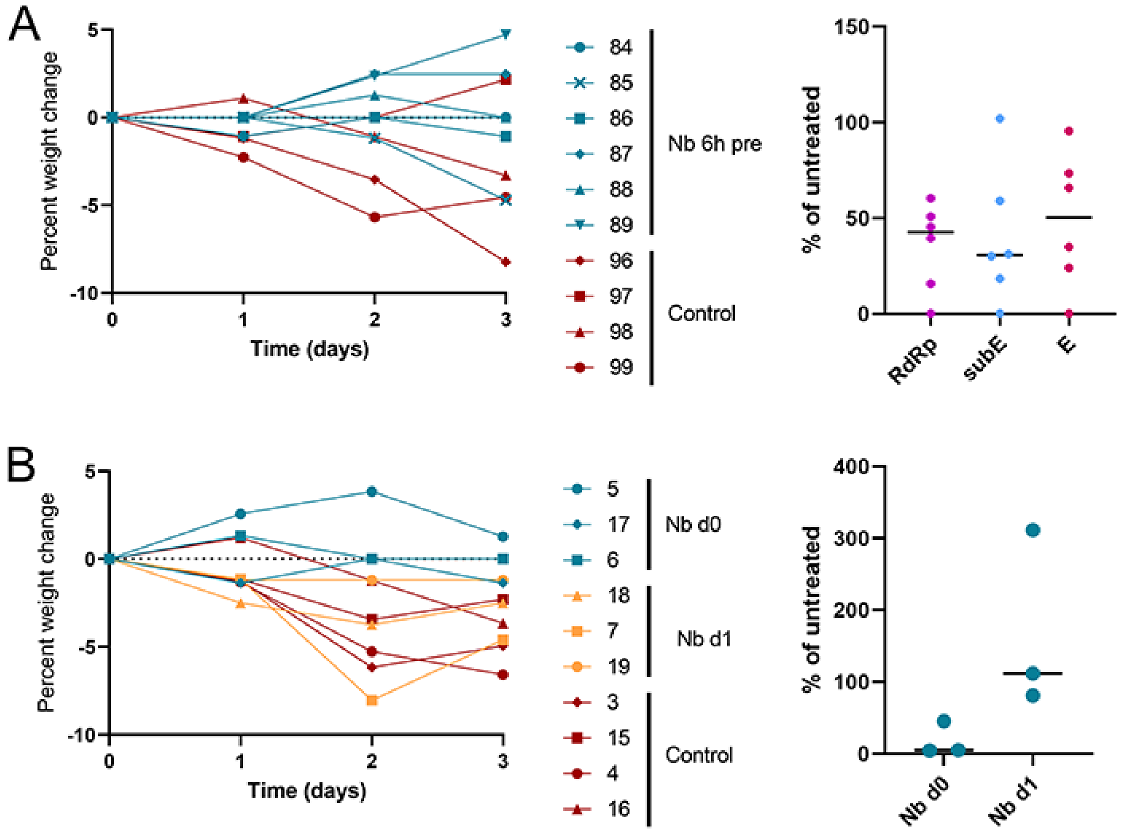

Figure S4. A) 35 µg Tri-TMH nanobody was given to hamsters intranasally six hours before infection, and SARS-CoV-2 RNA (RdRp, subE, and E) was quantified from lungs with RT-qPCR. Results are shown as the percentage of the average value for untreated individuals (n=6 in treated and n=4 in untreated hamsters). **B**) In a small pilot experiment (n = 3 per group), 20 µg Tri-TMH was administered intranasally to hamsters either at the same time as they were infected with SARS-CoV-2 (d0) or one day post infection (d1). SARS-CoV-2 RNA (N) was quantified with RT-qPCR from the d0 and d1 groups. Results are shown as the percentage of the average value for untreated individuals (n=4). Lines in all panels stand for the median. The body weight of the hamsters was recorded daily and the percentage weight change from baseline was plotted for each animal.

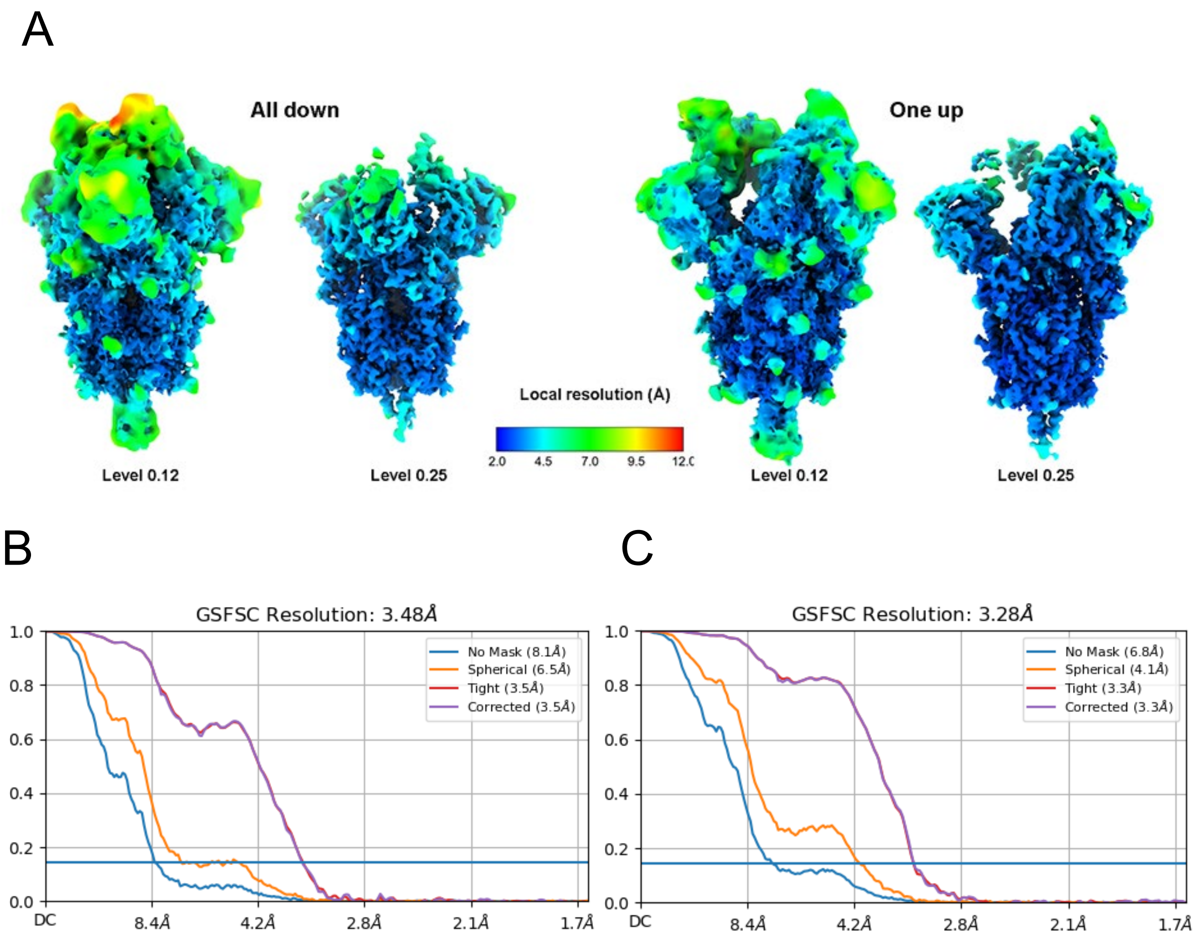

Figure S5. Resolution estimates of cryo-EM reconstructions. A) Local resolution was estimated and the maps were filtered to local resolution in cryoSPARC. The one-up and all-down maps are each shown at two volume levels. B-C) FSC curves for the all-down map (B) and the one-up map (C) from cryoSPARC.

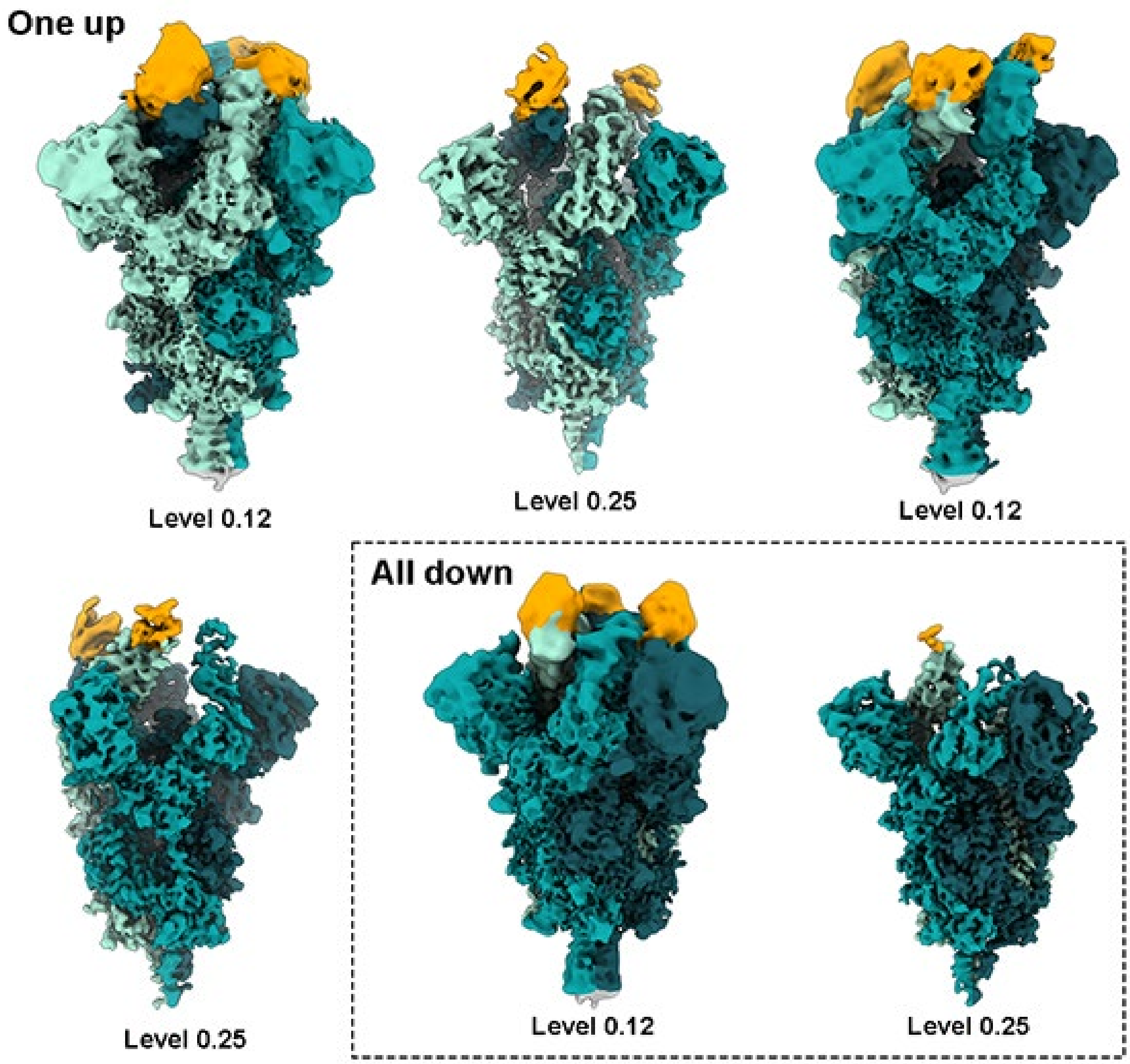

Figure S6. Nanobody-bound spike cryo-EM maps rendered at different contour levels. The one-up map (four views of the same map) and the all-down map are each shown at two different volume contour levels. At level 0.12, density corresponding to three bound nanobodies in observed, whereas level 0.25 allows the visualization of detailed density features in high resolution regions.

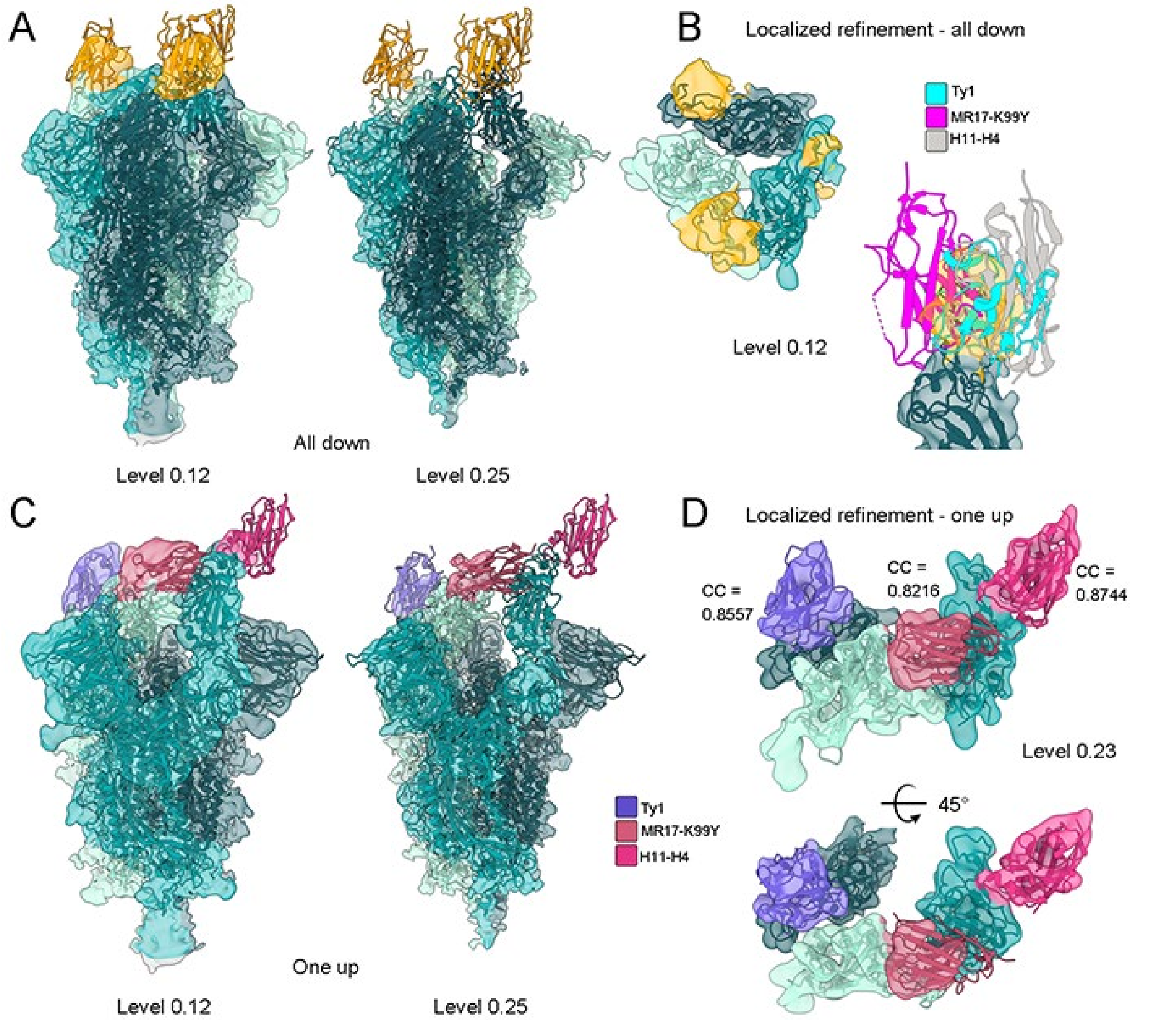

Figure S7. Fit of spike and nanobody PDB models in the cryo-EM maps. Model of the SARS-CoV-2 S trimer (PDB: 7A29) and models of nanobody-RBD complexes (PDB: 6ZHD, 6ZXN, 7CAN), displayed as ribbon representations, fitted into the cryo-EM maps. **A**) Map with all RBDs down. **B**) Localized refinement of each RBD-Nb region in the all-down (closed) conformation spike data did not improve nanobody density features, indicating a mixed population of different modules at each site in the reconstruction. Binding modes of the three nanobodies are shown and indicate that an average density of the three modules is likely to roughly coincide with the position of Ty1. Density features did not support the unambiguous identification of the nanobody modules in the reconstruction of the closed spike conformation. **C**) Map with one RBD up. **D**) Localized refinement of each RBD-Nb region in the partially open spike data improved nanobody density features and allowed the confident placement of each tri-TMH module using fitmap global search in ChimeraX. Cross-correlation scores of the top placement, fitting shown in panel D, were 0.86 for Ty1–RBD (from 6ZXN), 0.82 for MR17-K99Y–RBD (7CAN), and 0.87 for H11-H4–RBD.

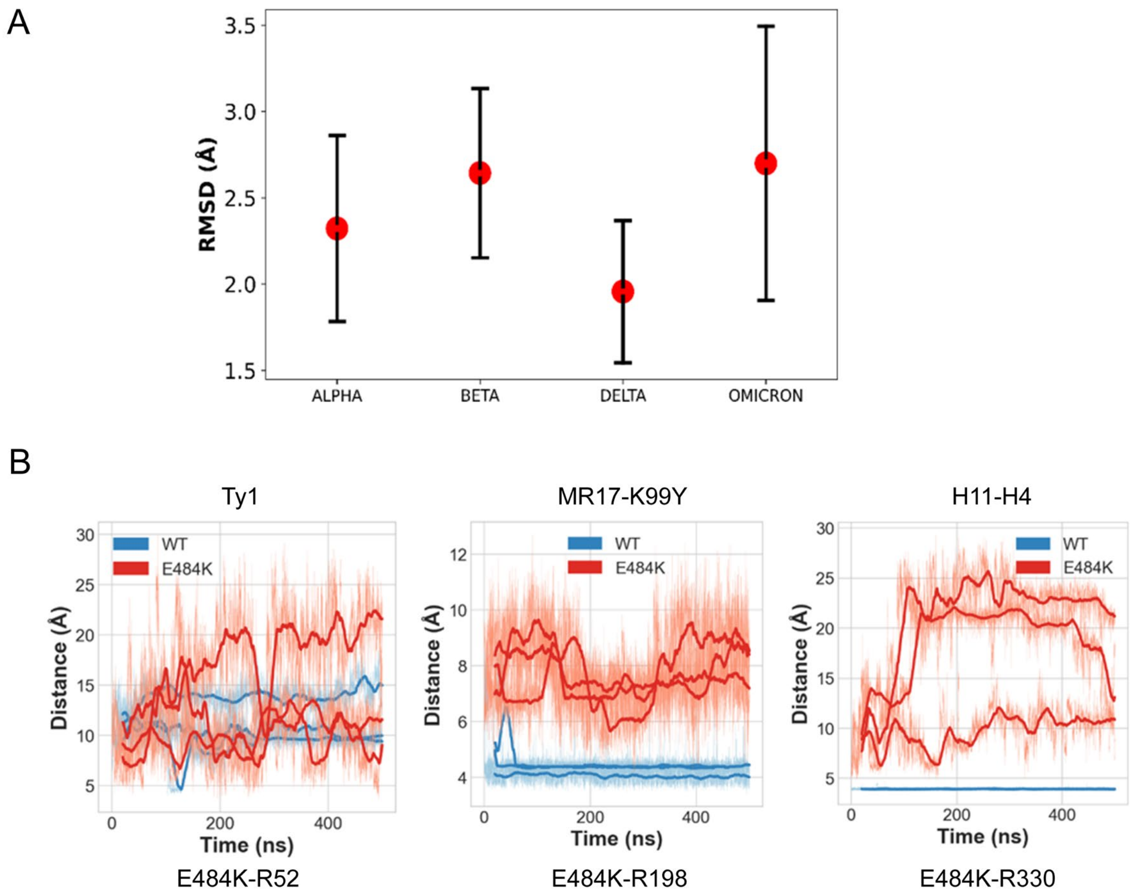

Figure S8. A) RMSD (Root Mean Square Displacement) of protein Cα atoms over MD simulation using the zero frame as a reference. Data from all simulation replicas are combined, and the mean value is shown as a dot, with the standard deviation as error bars. **B**) Distance between charged residues (E/K and R) in MD simulations. The three simulation replicas are shown separately for WT (blue) and E484K (red). The bold lines represent a running average of the previous 20 ns simulation data. Distances were measured between the glutamic acid CD atoms or lysine NZ atoms, and arginine CZ atoms.

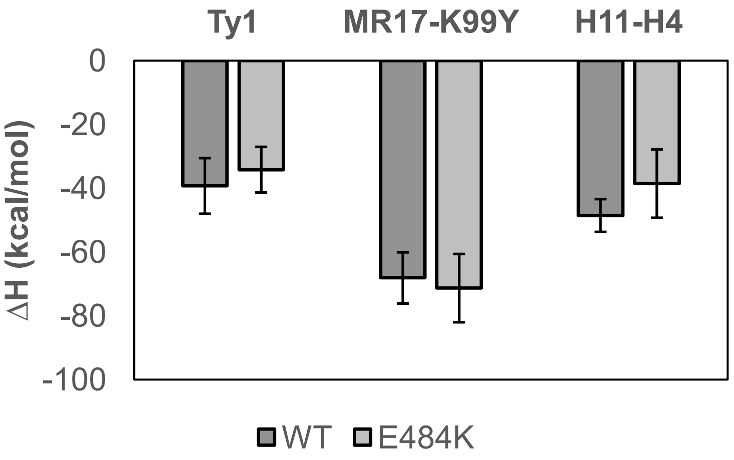

Figure S9. Binding enthalpy (ΔH) of each tri-TMH module and SARS-CoV-2 RBD. ΔH were determined by Molecular Mechanics Poisson-Boltzmann Surface Area (MMPBSA) calculations on the MD trajectories.

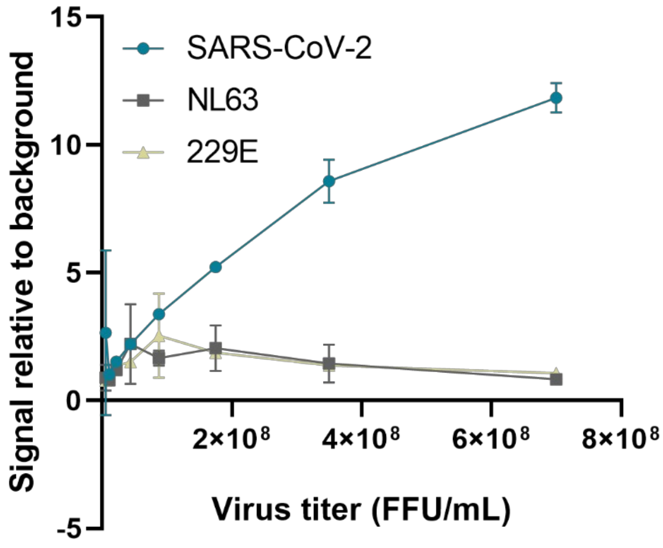

Figure S10. Detection of UV-inactivated SARS-CoV-2 using nanobodies fused with split nanoluciferase. Luciferase substrate and nanobodies fused with split nanoluciferase were mixed with UV-inactivated SARS-CoV-2, hCoV-NL63, or hCoV-229E. The resulting luminescence signal was measured from three replicates. Signal has been normalized against background (BSA). At titers above 1 × 10^8, UV-inactivated SARS-CoV-2 can be distinguished from background signal and other human coronaviruses.

Table S1. Reported binding and neutralization properties of nanobody modules.

**Nanobody**	**Citation**	**K_D (nM)**	**IC50 (nM)**
Ty1	(2)	5—10	54
H11-H4	(3)	12±1,5	6*
MR17-K99Y	(4)	33	38**
VHH V	(5)	8,92	142

*Neutralization efficiency reported for bivalent construct with human IgG Fc.

**Calculated based on the IC50 value of 0.50 μg/mL and the molecular weight of 13.9 kDa.

Table S2. Description of model systems and MD simulation time scales. Three replicas of 500 nanosecond simulations were run for each simulation setup.

Simulation setup n:o	Nanobody monomer	Mutations
**1**	Ty1	-
**2**	Ty1	E484K
**3**	MR17-K99Y	-
**4**	MR17-K99Y	E484K
**5**	H11-H4	-
**6**	H11-H4	E484K
**8**	H11-H4	**Alpha**: N501Y
**10**	H11-H4	**Beta**: K417N, E484K, N501Y
**12**	H11-H4	**Delta**: T478K, L452R
**14**	H11-H4	**Omicron**: G339D, S371L, S373P, S375F, K417N, N440K, G446S, S477N, T478K, E484A, Q494R, G496S, Q498R, N501Y, Y505H

Table S3. Cryo-EM data collection and processing statistics

	all-down map (J285)	1-up map (J275)
Magnification	165 000x	165 000x
Voltage (kV)	300	300
Electron exposure (e-/Å^2)	55.0	55.0
Defocus range (µm)	-1.5 to -3.0	-1.5 to -3.0
Pixel size (Å)	0.82	0.82
Symmetry imposed	None	None
Initial particle images (no.)	84049	84049
Final particle images (no.)	26708	42440
Map resolution (Å)	3.48	3.28
FSC threshold	0.143	0.143

Supplementary methods

Expression and purification of multimodular and luciferase-fused nanobodies. Nanobody expression cultures of *Escherichia coli* Rosetta-gami 2 (DE3) cells (Novagen) were grown at 37 °C) in autoinduction media until OD600 reached 0.5, after which the incubation temperature was lowered to 28 °C. The cells were harvested after 24 h from inoculation. To purify the proteins, bacterial cell pellets were resuspended in lysis buffer (10 mM Tris-HCl pH 7.5, 150 mM NaCl) with protease inhibitors and 10 mM imidazole. Cells were lysed with Emulsiflex C3, and the lysate was clarified by centrifugation at 38 000 × g for 30 min at 4 °C. Proteins were purified by immobilized nickel affinity chromatography with a 5 ml HisTrap FF crude column (Cytiva), using 300 mM imidazole for elution. Concentrated eluates were further purified by size-exclusion chromatography (SEC), using the ÄKTA Go system and a Superdex 75 Increase 10/300 column (Cytiva) in PBS buffer. For neutralization assays, 6xHis-tags were removed by enterokinase cleavage (Bovine enterokinase, GenScript). The cleaved tags and enzyme were removed from the sample using HisPur Ni-NTA resin (Thermo Scientific) or SEC as described above.

Expression and purification of recombinant SARS-CoV-2 S protein. The Expi293F™ (Thermo Fisher Scientific) suspension cells were grown at a density of 3×10^6 cells per ml using the ExpiFectamine™ 293 Transfection Kit (Thermo Fisher Scientific). Transfected cells were cultivated on an orbital shaker at 36.5 °C and 5% CO2 for six days, after which supernatant was harvested, clarified by centrifugation, filtered through a 0.45 μM filter, and supplemented with imidazole to 3 mM final concentration. SARS-CoV-2 S-protein was purified from the supernatant by immobilized nickel affinity chromatography with a 1-ml HisTrap excel column (Cytiva) using 300 mM imidazole for elution. The eluate was concentrated and buffer exchanged to 10 mM Tris pH 8 + 150 mM NaCl buffer, and S-trimer was used for cryo-EM grid preparation immediately after purification.

RT-qPCR. RNA was extracted from lung samples using Trizol (Thermo Scientific) according to the manufacturers' instructions. Isolated RNA was directly subjected to one-step RT-qPCR analysis based on a previously described protocol for RdRp (6) and for E and subE genes (7) with TaqMan fast virus 1-step master mix (Thermo Scientific) using AriaMx instrumentation (Agilent, Santa Clara, CA, USA). The actin RT-qPCR used for normalization is described in (8). Fold differences between samples were calculated by the comparative Ct method (9) using the average of normalized Ct values from non-nanobody treated infected animal lung tissues as reference.

Supplementary references

1. Emsley P, Lohkamp B, Scott WG, Cowtan K. 2010. Features and development of Coot. Acta Crystallogr D Biol Crystallogr 66:486-501.
2. Hanke L, Vidakovics Perez L, Sheward DJ, Das H, Schulte T, Moliner-Morro A, Corcoran M, Achour A, Karlsson Hedestam GB, Hällberg BM, Murrell B, McInerney GM. 2020. An alpaca nanobody neutralizes SARS-CoV-2 by blocking receptor interaction. Nature Communications 11:1-9.
3. Huo J, Mikolajek H, Le Bas A, Clark JJ, Sharma P, Kipar A, Dormon J, Norman C, Weckener M, Clare DK, Harrison PJ, Tree JA, Buttigieg KR, Salguero FJ, Watson R, Knott D, Carnell O, Ngabo D, Elmore MJ, Fotheringham S, Harding A, Moynié L, Ward PN, Dumoux M, Prince T, Hall Y, Hiscox JA, Owen A, James W, Carroll MW, Stewart JP, Naismith JH, Owens RJ. 2021. A potent SARS-CoV-2 neutralising nanobody shows therapeutic efficacy in the Syrian golden hamster model of COVID-19. Nature Communications 12.
4. Li T, Cai H, Yao H, Zhou B, Zhang N, van Vlissingen MF, Kuiken T, Han W, GeurtsvanKessel CH, Gong Y, Zhao Y, Shen Q, Qin W, Tian XX, Peng C, Lai Y, Wang Y, Hutter CAJ, Kuo SM, Bao J, Liu C, Wang Y, Richard AS, Raoul H, Lan J, Seeger MA, Cong Y, Rockx B, Wong G, Bi Y, Lavillette D, Li D. 2021. A synthetic nanobody targeting RBD protects hamsters from SARS-CoV-2 infection. Nat Commun 12:4635.
5. Koenig P-A, Das H, Liu H, Kümmerer BM, Gohr FN, Jenster L-M, Schiffelers LDJ, Tesfamariam YM, Uchima M, Wuerth JD, Gatterdam K, Ruetalo N, Christensen MH, Fandrey CI, Normann S, Tödtmann JMP, Pritzl S, Hanke L, Boos J, Yuan M, Zhu X, Schmid-Burgk JL, Kato H, Schindler M, Wilson IA, Geyer M, Ludwig KU, Hällberg BM, Wu NC, Schmidt FI. 2021. Structure-guided multivalent nanobodies block SARS-CoV-2 infection and suppress mutational escape. Science 6230:eabe6230.
6. Corman VM, Landt O, Kaiser M, Molenkamp R, Meijer A, Chu DK, Bleicker T, Brunink S, Schneider J, Schmidt ML, Mulders DG, Haagmans BL, van der Veer B, van den Brink S, Wijsman L, Goderski G, Romette JL, Ellis J, Zambon M, Peiris M, Goossens H, Reusken C, Koopmans MP, Drosten C. 2020. Detection of 2019 novel coronavirus (2019-nCoV) by real-time RT-PCR. Euro Surveill 25.

7. Dagotto G, Mercado NB, Martinez DR, Hou YJ, Nkolola JP, Carnahan RH, Crowe JE, Baric RS, Barouch DH, Pfeiffer JK. 2021. Comparison of Subgenomic and Total RNA in SARS-CoV-2-Challenged Rhesus Macaques. Journal of Virology 95:e02370-20.
8. Zivcec M, Safronetz D, Haddock E, Feldmann H, Ebihara H. 2011. Validation of assays to monitor immune responses in the Syrian golden hamster (Mesocricetus auratus). J Immunol Methods 368:24-35.
9. Schmittgen TD, Livak KJ. 2008. Analyzing real-time PCR data by the comparative CT method. Nature Protocols 3:1101-1108.
